# An AFM-Based Stiffness Clamp for Dynamic Control of Rigidity

**DOI:** 10.1371/journal.pone.0017807

**Published:** 2011-03-08

**Authors:** Kevin D. Webster, Ailey Crow, Daniel A. Fletcher

**Affiliations:** 1 Biophysics Graduate Group, University of California, Berkeley, California, United States of America; 2 Department of Bioengineering, University of California, Berkeley, California, United States of America; 3 Physical Biosciences Division, Lawrence Berkeley National Laboratory, Berkeley, California, United States of America; Clarkson University, United States of America

## Abstract

Atomic force microscopy (AFM) has become a powerful tool for measuring material properties in biology and imposing mechanical boundary conditions on samples from single molecules to cells and tissues. Constant force or constant height can be maintained in an AFM experiment through feedback control of cantilever deflection, known respectively as a ‘force clamp’ or ‘position clamp’. However, stiffness, the third variable in the Hookean relation *F = kx* that describes AFM cantilever deflection, has not been dynamically controllable in the same way. Here we present and demonstrate a ‘stiffness clamp’ that can vary the apparent stiffness of an AFM cantilever. This method, employable on any AFM system by modifying feedback control of the cantilever, allows rapid and reversible tuning of the stiffness exposed to the sample in a way that can decouple the role of stiffness from force and deformation. We demonstrated the AFM stiffness clamp on two different samples: a contracting fibroblast cell and an expanding polyacrylamide hydrogel. We found that the fibroblast, a cell type that secretes and organizes the extracellular matrix, exhibited a rapid, sub-second change in traction rate (*dF/dt*) and contraction velocity (*dx/dt*) in response to step changes in stiffness between 1–100 nN/µm. This response was independent of the absolute contractile force and cell height, demonstrating that cells can react directly to changes in stiffness alone. In contrast, the hydrogel used in our experiment maintained a constant expansion velocity (*dx/dt*) over this range of stiffness, while the traction rate (*dF/dt*) changed with stiffness, showing that passive materials can also behave differently in different stiffness environments. The AFM stiffness clamp presented here, which is applicable to mechanical measurements on both biological and non-biological samples, may be used to investigate cellular mechanotransduction under a wide range of controlled mechanical boundary conditions.

## Introduction

Atomic force microscopy (AFM), initially developed as a topographical imaging modality, has become an important tool for investigating the mechanical properties and dynamic behavior of biological molecules, materials, cells, and tissues [1]. AFM-based techniques in cell and molecular biology leverage the high resolution of AFM in space, time, and force to study properties such as cell adhesion mechanics [2], polymer network dynamics [3], and protein folding [4]. Here we present the development of a method for dynamically varying AFM cantilever stiffness that takes advantage of precise AFM feedback control to create changes in the external rigidity felt by active samples. We use this method, which we call a ‘stiffness clamp’ by analogy to the existing ‘force clamp’ and ‘position clamp’, to investigate the cellular response to rigidity.

The rigidity of the cellular microenvironment has been shown to be an important input signal that influences a range of biological processes [5]. The resistance to deformation of tissues in vivo, characterized by an elastic modulus, varies from near 100 pascals for soft tissues such as the brain to tens of thousands of pascals for muscle tissue and up to millions of pascals for cartilage. This tissue rigidity, or stiffness, serves as an important in vivo cue in processes such as embryogenesis [6], cell proliferation [7], and angiogenesis [8]. Notably, numerous experiments have demonstrated the influence of microenvironmental rigidity in vitro on cellular morphology [9], motility [10], and differentiation [11]. While the importance of stiffness has been well-documented, the dynamics of rigidity sensing are poorly understood.

The predominant methods for studying the effects of microenvironmental rigidity on cellular behaviors involve culturing cells on deformable substrates (e.g. thin rubber films [12], polyacrylamide hydrogels [13], and microfabricated posts [14]). These studies, while instrumental in establishing the effect of substrate rigidity on cellular behaviors, are limited to a single static rigidity for each experiment. Similarly, the spring-like behavior of optical traps, AFMs, and microplates has also been used to expose single cells to different microenvironmental rigidities but these usually use only a single rigidity per experiment [15]–[17]. To expose a given cell to multiple rigidities, some studies have employed static rigidity gradients [18], [19] or substrates of anisotropic rigidity [10]. Recent studies have demonstrated hydrogels with dynamic rigidities that utilize UV exposure [20] or DNA crosslinking [21] to change rigidity mid-experiment, though the stiffness changes are relatively slow, not reversible, and can only sample a narrow range of elastic moduli. Furthermore, none of these techniques distinguish between the cell's response to force, deformation, and stiffness. Recently, a custom-built parallel microplate system was used in combination with double-feedback to change the effective stiffness experienced by a single cell spread between the microplates [22]. While AFMs have the advantage of high resolution in space, time, and force, and cells can spread between a microfabricated cantilever and a surface [16], [23], AFM systems are currently limited to a single stiffness per experiment given by the native cantilever stiffness.

We have developed an AFM feedback algorithm to reversibly and rapidly change the stiffness presented to the sample while accurately measuring force and deformation. We apply this AFM stiffness clamp to study the dynamics of an expanding hydrogel and a single cell in response to step changes in stiffness.

## Results

### Stiffness clamp concept

**Figure 1:**
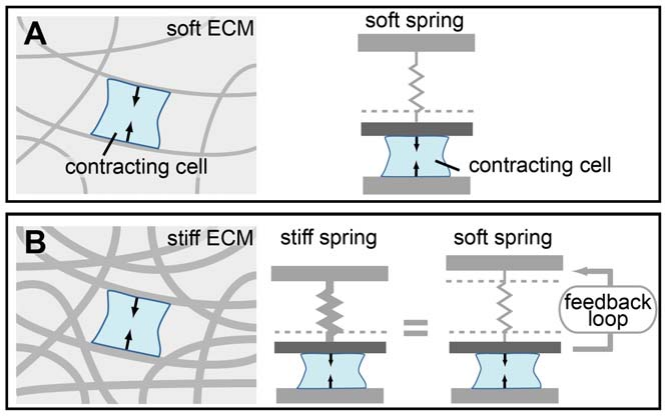
Feedback control can change the apparent stiffness a cell experiences. (a) A contracting cell in a soft extracellular matrix (ECM) experiences little resistance to its contraction and can be modeled with a soft spring. (b) A contracting cell in a stiff ECM experiences a large resistance to its contraction and can be modeled with a stiff spring. Using the AFM stiffness clamp, a soft spring can be made to appear stiff (or vice-versa) by controlling the spring's extension as a function of the cell's contraction. This approach can be broadly applied to make springs appear stiffer or softer than their actual value.

The mechanical interaction of contractile cells with their microenvironment, which is composed of polymeric extracellular matrix (ECM) proteins and other cells, can be modeled most simply as a cell pulling on a spring (FIG. 1A). Setting aside the nonlinear behavior of the ECM temporarily, a cell that deforms a Hookean spring experiences a resistance force given by the spring constant and the amount of deformation. The goal of the stiffness clamp is to tune the apparent stiffness a cell experiences by controlling how much force the cell must exert to change its height a given amount through feedback control of the spring deflection (FIG. 1B).

**Figure 2:**
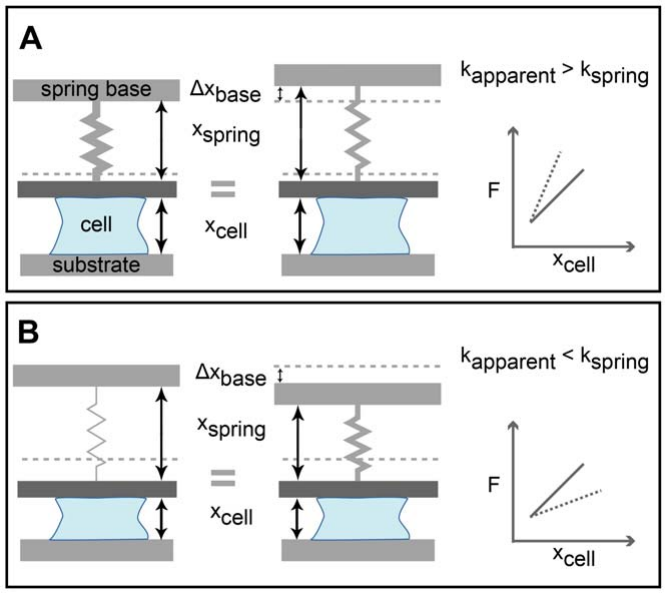
Conceptual design of the AFM stiffness clamp. (a) A stiff spring can be simulated using a spring of a smaller stiffness. A cell applying a given force against a stiff spring achieves a smaller change in height than a softer spring. Moving the spring base up as the cell contracts makes a softer spring appear stiffer to the contracting cell. Plotting contractile traction force 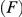 versus cell height 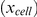 produces a trace whose steep slope is the apparent stiffness, 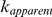 (dotted line) and is greater than the native spring stiffness, 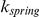 (solid line). (b) A soft spring can be simulated using a spring of a greater stiffness. A cell applying a given force against a soft spring achieves a greater change in height than a stiffer spring. Moving the spring base down as the cell contracts makes a stiffer spring appear softer to the contracting cell. Plotting traction force 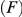 versus cell height 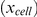 produces a trace whose gradual slope is the apparent stiffness, 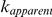 (dotted line) and is less than the native spring stiffness, 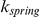 (solid line).

In theory, a wide range of apparent stiffnesses may be achieved using only a single spring together with feedback control (FIG. 2). If the spring base is moved away from the cell as it contracts, the spring will appear stiffer to the contracting cell than it actually is (FIG. 2A). If the spring base is moved upwards, away from the cell by the same amount that the cell deflects the spring downward, then the cell height, 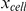, will remain constant. Given this constraint, regardless of the force the cell exerts on the spring, the cell's height does not change, thereby exposing the cell to an infinitely stiff microenvironment 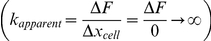. By moving the spring base toward the cell as it contracts, the spring will appear softer than it actually is (FIG. 2B). If the feedback routine moves the spring base such that the spring does not change in length, the force exerted on the spring remains constant, and the stiffness of the microenvironment appears to be infinitely soft 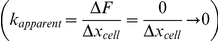. These two limits of constant height and constant force have been used elsewhere and are known as the position and force clamp, respectively [24]. Force and position clamps are based on a simple PID-feedback routine that uses the error between a given setpoint force or position and the current force or position to adjust the sample position. In contrast, stiffness is defined as the *change* in force over the *change* in displacement and therefore cannot be controlled using conventional feedback routines. The AFM stiffness clamp presented here is able to dynamically tune apparent stiffness *between* the extremes of infinitely soft and stiff.

### Stiffness clamp applied to an expanding hydrogel

**Figure 3:**
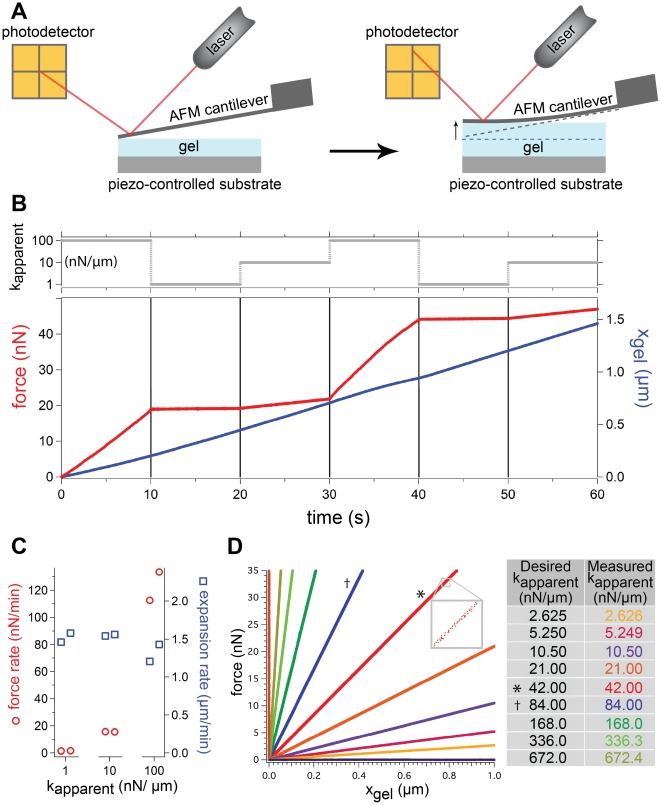
Response of expanding hydrogel to step changes in stiffness. (a) The AFM stiffness clamp was applied to a rehydrated hydrogel that deflected an AFM cantilever as it expanded. Cantilever position is precisely measured using an optical lever system. Feedback was implemented by moving a piezo-controlled substrate. (b) A typical trace of how force and gel height (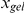) changed over time as the cantilever deflected in response to the expansion of the hydrogel against apparent stiffnesses of 1, 10, and 100 nN/µm. Separate experiments conducted on 5 different gels all exhibited the same stiffness-dependent behavior shown above. Note that the slope of the force trace clearly changes when the apparent stiffness changes, while the slope of the height trace remains basically constant over this range of stiffness. (c) Categorical plot of the force rate and velocity of gel expansion under three different apparent stiffnesses from the trace depicted in (b). The rates are determined from a linear regression fit where the 95% confidence interval for each slope is within 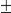
0.25 nN/min and 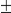
5 nm/min for the force and height, respectively. Force rate changes with stiffness while expansion rate does not over this range of stiffness. (d) Plot of force 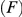 versus gel height 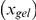 as the gel expanded under a wide range of apparent stiffnesses. Each trace represents a different apparent stiffness listed in the table and applied using the stiffness clamp algorithm. The traces were translated to begin at the origin for comparison. The horizontal and vertical traces represent desired stiffnesses approaching 0 and 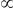, corresponding to a force and position clamp with standard deviations of 15 pN and 0.34 nm. Inset depicts the discrete but highly linear nature of the data. The * marks the trace without any feedback loop and whose slope is the spring constant of the cantilever, 42 nN/µm.

We tested the ability of the AFM stiffness clamp algorithm to produce a range of apparent stiffnesses with an expanding hydrogel, and we characterized the material's response to step changes in stiffness. Addition of phosphate buffered saline (PBS) to a dehydrated ∼1 kPa polyacrylamide hydrogel caused it to gradually expand. As the gel expanded and increased in height, it pushed against the cantilever applying an increasing force (FIG. 3A&B). Without the stiffness clamp feedback loop, the spring constant of the cantilever defined how much force the gel applied to increase its height. When we changed the apparent stiffness of the cantilever using the stiffness clamp between 1–100 nN/µm, there was an immediate change in the force rate due to the modified feedback control of the cantilever position, while the gel expansion rate remained essentially constant (FIG. 3C). This behavior was observed for N = 5 gels.

With a single AFM cantilever with spring constant 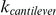, we used the stiffness clamp to apply 11 different stiffnesses ranging from 0 to infinity as the gel expanded. By plotting the cantilever force versus the gel height we obtained a series of traces where the slopes define the achieved apparent stiffness (FIG. 3D). The apparent stiffness measured from the slope of the traces in FIG. 3D was less than 0.1% different from the desired value for a range of stiffnesses spanning two orders of magnitude from 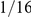 to 16 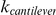. The most extreme apparent stiffnesses (force clamp and position clamp) produced traces with Gaussian noise around a constant force and height with standard deviations of 15 pN and 0.34 nm, respectively. (See supporting file 1 for further information.) Figure 3D demonstrates that we can accurately apply a wide range of apparent stiffnesses on an expanding hydrogel, all with a single cantilever, using the AFM stiffness clamp.

### Stiffness clamp applied to a contracting cell

**Figure 4:**
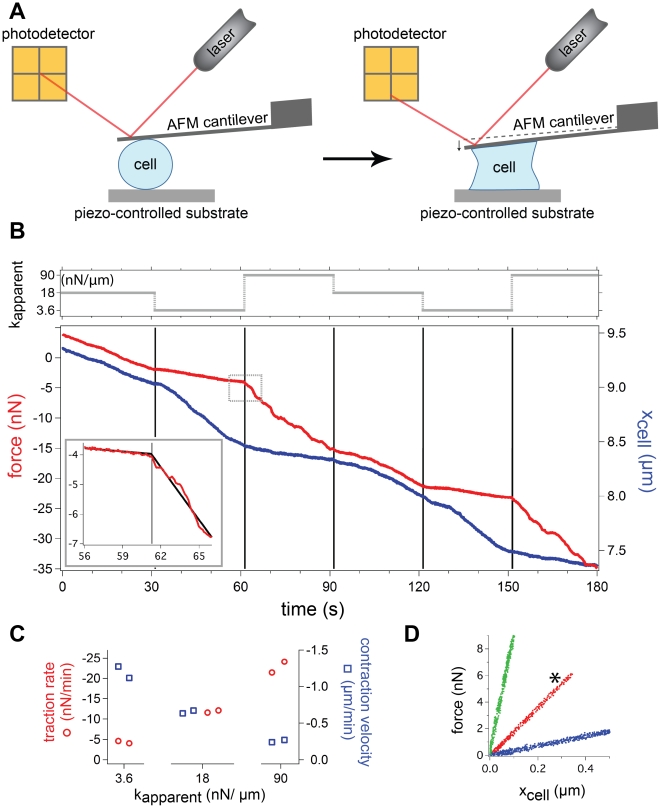
Cell contraction rapidly responds to stiffness changes. (a) An AFM was used to expose a single fibroblast cell to dynamically changeable apparent stiffness values with the AFM stiffness clamp. The piezo-controlled substrate was moved in response to deflections of the cantilever, which were precisely measured with an optical lever system. (b) Force and cell height as the cell contracts under different apparent stiffnesses from a typical experiment. A total of 30 cells were tested, all exhibiting the same stiffness-dependent behavior shown above. Each interval is under an apparent stiffness of 3.6, 18, or 90 nN/µm as indicated at the top of the graph. The traction rate and contraction velocity rapidly change with a step change in stiffness. A segmented linear regression fit is plotted highlighting the change in traction rate (inset). Data displayed in (c) and (d) are compiled from this trace. (c) Traction rate increases with apparent stiffness while corresponding contraction velocity decreases. The rates are determined from a linear regression fit where the 95% confidence interval for each slope is within 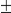
0.4 nN/min and 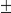
20 nm/min for the force and height, respectively. (d) Plot of force versus cell height. The three linear, distinct traces each have slopes that indicate that the desired apparent stiffnesses were achieved. The * marks the trace without any feedback loop. Each interval was translated to begin at the origin for comparison.

Fibroblast cells are used extensively as a model system to investigate the effect of substrate rigidity [5], [9], [13], [14], [18]. After demonstrating the range and precision of the stiffness clamp algorithm with a hydrogel, we used NIH 3T3 fibroblast cells to investigate how cellular rigidity sensing responds to a reversible step change in stiffness. Figure 4 shows the results of a typical experiment. Cells in suspension were flowed into a chamber and within minutes were brought into contact with both a fibronectin-coated glass surface and a fibronectin-coated tipless AFM cantilever 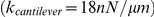. After a small compressive force 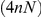 established contact, adhesions formed on both surfaces, and the cell contracted (FIG. 4A). Once contraction started we cycled between stiffnesses of 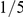, 1, and 5 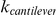 (3.6, 18, 90 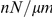) every 30 s. We chose a cycle period of 30 s to allow for exchange of cytoskeletal and focal adhesion components (timescale of seconds) but not full reorganization of adhesions or the cytoskeleton (timescale of minutes) [25]. A typical resulting traction force and cell height trace is shown in FIG. 4B.

We found that when the apparent stiffness changed to a larger value, the cell's traction rate 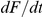 rapidly increased while the corresponding contraction velocity 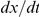 decreased (FIG. 4D). Notably, this change in traction rate and contraction velocity happens nearly instantaneously (within 0.5 s) (FIG. 4B inset), indicating that cells can reversibly respond to a stiffness cue on a whole cell level on a timescale of seconds. The stiffness-dependent traction rate and velocity were found to be reversible and consistent for a given cell, despite changes in absolute cell height and contractile force. Even though the absolute cell tension was greater later during contraction, the traction rate was dependent only on the instantaneously applied stiffness (and similarly for cell height and contraction velocity). Importantly, this indicates that the response of contraction rate is specifically due to a change in stiffness and not the cell tension or height. This behavior was observed for N = 30 cells.

## Discussion

The AFM stiffness clamp provides a high-resolution method for varying apparent stiffness and evaluating cellular responses including contraction behavior. Using the AFM stiffness clamp, we show that cells rapidly change their traction rate and contraction velocity in response to step changes in apparent stiffness. Importantly, the stiffness clamp algorithm dynamically changes the apparent stiffness while the force and height are unchanged in the instant before and after the stiffness change. Therefore, any cellular response is a function of the step change in stiffness and not force or height. This decoupling of stiffness from force and height unambiguously shows that stiffness changes alone caused the change in contraction.

Our observation of stiffness dependent contraction of single cells is consistent with several previous studies. We recently used the high-resolution of AFM to characterize the contraction dynamics of single human platelet cells [16] and found that the force generation of platelets was dependent on microenvironmental stiffness, though each platelet was exposed to only a single stiffness. Other techniques, using systems limited to a single stiffness per experiment, have also observed a dependence of contraction on stiffness with a variety of cell types [10], [15], [19]. Our results with the AFM stiffness clamp are consistent with a recent study by Mitrossilis et al. that used a custom-built parallel microplate system to change the stiffness experienced by a single myoblast cell and found that traction rate was higher for larger stiffnesses and did not depend on absolute force [22].

It is worthwhile to note that the AFM stiffness clamp presented here only alters stiffness in one axis, though as demonstrated above, this appears to be sufficient to elicit a response from the contracting cell. Due to the fact that stiffness can only be measured by displacing a sample, the apparent stiffness can only be applied when cell height is actively changing, for example during fibroblast contraction, cardiomyocyte beating, neutrophil shape change in response to chemoattractants, and cell rounding during mitosis.

This AFM-based approach to dynamically tuning microenvironmental rigidity is broadly applicable to both biological and non-biological experimental situations. In essence, the algorithm we present can be applied to any system with a spring where there is precise knowledge of the force and a single means of adjusting the position of the spring base (as illustrated in FIG. 2). This stiffness clamp algorithm has the advantage of requiring only one actuator and therefore can be used with existing commercial AFMs. Furthermore, the algorithm can be adjusted to emulate nonlinear elastic properties, such as those of specific ECM networks.

In the case of single molecule experiments on mechanosensitive molecules, which typically employ an AFM or optical trap [26], the AFM stiffness clamp could be implemented to sample a wide range of apparent stiffness values. The stiffness clamp can also be integrated with cell rheology measurements and fluorescence microscopy to characterize the viscoelastic properties of the cell and protein localization under various apparent stiffnesses. At the multicellular scale, tissue stiffness has been shown to affect the cancerous phenotype of cell colonies [27], and the AFM stiffness clamp could be used to study the responses of tissues in microenvironments of changing stiffness. Importantly, our system allows for the use of apparent stiffness values outside of those that can be achieved by standard cantilever fabrication methods.

In this study, we have presented an AFM-based method for dynamically changing the apparent stiffness of the microenvironment surrounding a cell. We demonstrated the high temporal and spatial resolution of the AFM stiffness clamp using an expanding hydrogel and contracting cell, finding that the cell contraction rate reversibly changes nearly instantaneously with stiffness and does not depend on absolute force or cell height. Both cellular traction rate and contraction velocity were stiffness-dependent, whereas the expansion velocity of the hydrogel used in our experiments remained constant for stiffnesses ranging 1–100 nN/µm. The AFM stiffness clamp provides a powerful tool for investigating the role of mechanical boundary conditions on cellular behavior.

## Materials and Methods

### Stiffness clamp algorithm

The AFM stiffness clamp is implemented using a feedback algorithm based on the extension of a Hookean spring 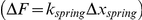, though this analysis can be extended to nonlinear springs. The microenvironmental stiffness a cell experiences is given by the amount of force it must apply to change its height, 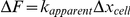. If the base of the spring can move by an amount 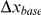, the change in cell height is given by the difference between spring extension and movement of the spring base, 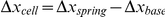. The force resisting the change in cell height is provided solely by the extension of the spring. Therefore, equating the expressions for 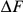 and solving for the movement of the spring base gives
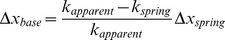
(1)which defines how much the base must be moved to achieve the desired apparent stiffness, 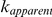, for a given deformation of the spring. Note that the position clamp can be obtained from Eq. (1) when 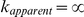, in which case the base moves the same amount as the spring deforms, and the cell height remains constant. Similarly, the force clamp results when 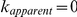 and 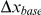
cancels out the movement of the spring, such that 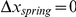.

The AFM stiffness clamp feedback algorithm uses the desired apparent stiffness 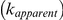, the spring stiffness 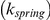, and Eq. (1), together with a measure of how much the cell deforms the spring, to determine how far to move the base. Equation (1) is directly used in the feedback algorithm for 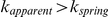, but for 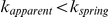 Eq. (1) grows out of bounds as 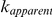 approaches zero. For 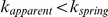, we alter Eq. (1) so that it iteratively converges to the same ratio 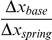 without growing out of bounds according to
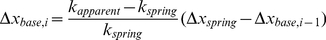
(2)where 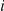 is the index for each cycle of the iteration and 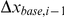 is the amount the base was moved in the previous iteration (see supporting file 1 for a detailed derivation).

### Atomic force microscope

Atomic force microscope (AFM) experiments were conducted using a modified Veeco Bioscope I mounted on a Zeiss Axiovert 25 inverted microscope. The Bioscope I z-axis piezo in our system has a range of only 4 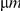. Since a larger z range is more convenient for working with cells, the substrate was moved instead of the cantilever base with a feedback-controlled Mad City Labs piezo-actuator stage and controller with a range of 50 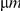 and a resolution of 0.1 nm. Cantilever deflection and substrate position was controlled with a National Instruments 16-bit, 250 kS/s PCI-6229 digital I/O card and a custom LabVIEW program to implement the stiffness clamp algorithm running at 100 Hz. The substrate was mounted on a heated stage and maintained at 37°C for cell experiments. Tipless silicon nitride MLCT (30–50 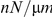, Veeco) and Arrow cantilevers (10–20 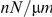, Nanoworld) were used for the gel and cell experiments, respectively. Calibration of the optical lever was conducted before each experiment by ramping a glass coverslip substrate up and down while in contact with the cantilever. The surface was ramped 450 nm and the average of 15 cycles was used to determine the volts to meters conversion factor. See supporting file 1 for a discussion on the effect of calibration errors on the apparent stiffness applied by the stiffness clamp. We then determined the cantilever spring constant before each experiment by recording the thermal fluctuations of the cantilever out of contact in air and fitting the first resonance peak of the power spectra with a Lorentzian function using the equipartition theorem [28]. This indicates that the resolution of the detection of the cantilever position was thermally limited.

To monitor drift in both the cell and gel experiments, we placed the cantilever in contact with the glass substrate in force clamp mode, immediately before each experiment. Experiments were not started until the system had equilibrated, such that a force clamp could be maintained with no significant change in stage position (generally 10–60 minutes). Drift over the course of the experiment was measured in two ways. First, the zero deflection point of the cantilever was compared before and after each experiment to measure any cantilever drift. Second, for cell experiments, the contact point between the surface and cantilever was measured before and after each experiment. These measurements confirmed that the drift over the course of the experiment was negligible compared to the active contraction of the cell and expansion of the gel. Drift accounted for <10% of the total deflection for all experiments used.

### Polyacrylamide hydrogels

The ∼1 kPa polyacrylamide hydrogel was dehydrated at 4°C overnight and was rehydrated immediately before the AFM experiment with a standard phosphate buffered saline (PBS) solution. The cantilever was brought into contact as the gel rehydrated and expanded.

### Cell culture

NIH 3T3 fibroblast cells were cultured in DMEM (GIBCO) supplemented with 10% fetal bovine serum, 100 U/ml penicillin, and 100 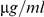 streptomycin. Cells were maintained in an incubator at 37°C with a humid, 5% CO_2_ atmosphere. A trypsin solution was used to detach cells at which point trypsin neutralizer was added and cells were then centrifuged at 300 g for 5 minutes. The resulting supernatant was discarded and cells were resuspended in their culture medium (DMEM plus supplements). KOH cleaned glass substrates and cantilevers were immersed for 30 min in a 50 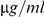 fibronectin solution (F0895, Sigma). The fibronectin solution was then washed off and cells were added and the cantilever was brought on top of a cell as it settled on the substrate.

### Statistical analysis

The inset of the FIG. 4B demonstrates the rapid change in traction rate upon a change in apparent stiffness. We found that this change occurred within 0.5 s. This response time was calculated by comparing two models with an F test with P values<0.01. First, a 30 s window was applied centered on the timepoint when 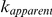 was changed. Then a simple linear regression was compared with a segmented linear regression where the timepoint of the intersection of the two segments must be determined from the data. This 30 s window was then moved earlier in time and the two models were again compared. The point at which the preferred model shifted to the simple linear regression is defined as the point when the traction rate has statistically changed.
